# α-Linolenic acid prevents endoplasmic reticulum stress-mediated apoptosis of stearic acid lipotoxicity on primary rat hepatocytes

**DOI:** 10.1186/1476-511X-10-81

**Published:** 2011-05-18

**Authors:** Yong Zhang, Lei Dong, Xia Yang, Hongyang Shi, Li Zhang

**Affiliations:** 1 Department of Gastroenterology, the Second Affiliated Hospital of Xi'an Jiaotong University, No. 157, West 5th Road, Xi'an, Shaanxi Province-710004, China; 2 Department of Respiration, the Second Affiliated Hospital of Xi'an Jiaotong University, No. 157, West 5th Road, Xi'an, Shaanxi Province-710004, China

## Abstract

**Aims:**

Lipid accumulation in non-adipose tissues leads to cell dysfunction and apoptosis, a phenomenon known as lipotoxicity. Unsaturated fatty acids may offset the lipotoxicity associated with saturated fatty acids. Stearic acid induced endoplasmic reticulum (ER) stress and caused apoptotic and necrotic cell death in the primary rat hepatocytes.

**Methods:**

Cell viability was investigated using MTT assay, and apoptosis was evaluated with Hoechst 33342 staining. Western blot analysis was used to examine the changes in the expression levels of glucose regulated protein 78 (GRP78), glucose regulated protein 94 (GRP94), and C/EBP homologous protein (CHOP). Caspase-3 activity was evaluated using a Caspase-3 substrate kit.

**Results:**

We have studied the ability of α-linolenic acid to prevent endoplasmic reticulum stress-mediated apoptosis of rat hepatocytes elicited by stearic acid and thapsigargin. Incubation of primary rat hepatocytes for 16 h with stearic acid produced a significant increase in cell death. Stearic acid also increased levels of three indicators of ER stress -- GRP78, CHOP, and GRP94. α-Linolenic acid distinctly reduced cell death and levels of all three indicators of ER stress brought about by stearic acid. Thapsigargin, which induces ER stress produced similar effects to those obtained using stearic acid; its effects were partly reversed by α-linolenic acid.

**Conclusion:**

These results suggest that α-linolenic acid prevents ER stress-mediated apoptosis of stearic acid lipotoxicity on primary rat hepatocytes might become a target to develop new antiapoptotic compounds in nonalcoholic fatty liver disease (NAFLD).

## 1. Introduction

The endoplasmic reticulum (ER) is a subcellular organelle where the vast majority of secreted and membrane proteins are folded. Many kinds of cellular perturbations, such as imbalances in calcium, loss of the luminal oxidizing environment and/or nutrient homeostasis, can lead to the accumulation of unfolded proteins and apoptosis[1,2]. The apoptosis is involved in the pathogenesis of many diseases and conditions including ischemia-reperfusion injury, diabetes and nonalcoholic fatty liver disease (NAFLD) [3,4].

Hepatocyte death is a main feature of almost every liver disease, with apoptosis characterized by biochemical and morphological features being one of its modes. Over the past ten years, thapsigargin (TG), has become widely used as selective inhibitor of the ubiquitous sarco-endoplasmic reticulum Ca^2+^-ATPases (SERCAs), which pump Ca^2+ ^from the cytosol into the lumen of the endoplasmic reticulum (ER) in mammalian cells [5-7] The latter mechanism of TG action has been described in detail during the last twenty years [8]. It has long been known that elevation of intracellular free calcium ([Ca^2+^]i) has cytotoxic consequence in many cells including hepatocytes [9]. ER function is mediated, in part, by intraluminal Ca^2+^-binding proteins, which include the glucose-regulated proteins GRP78 and GRP94 [10,11].

Several studies have linked ER dysfunction and activation of the unfolded protein response (UPR) to impairments in glucose homeostasis and diabetes [12,13]. ER stress and activation of the UPR in the liver have also been observed in genetic and dietary murine models of obesity, dietary models of NAFLD, and in humans with NAFLD [13-15]. A large portion of the elevated hepatic triglyceride stores in NAFLD appear to arise from re-esterification of circulating free fatty acids [16]. Elevated circulating free fatty acids are positively correlated with liver disease severity in individuals with NAFLD [17]. Increased non-esterified fatty acids, in particular long chain saturated fatty acids, induce ER stress and activate the UPR in a number of cell types, including hepatocytes [15,18,19].

The metabolic signals that elicit ER stress are still poorly defined. Recent studies imply that long-chain saturated fatty acids, may induce ER stress in breast cancer cells and liver cells [15,3,20]. Elevated lipids also induce apoptosis in many cell types, thus ER stress and an inability of the UPR to reestablish ER homeostasis may be upstream components of lipotoxicity [20,21]. Therefore, impairments in ER homeostasis and the UPR have been implicated in a number of disease states including obesity, diabetes, hepatitis C, and NAFLD.

The present study had three aims: (1) To examine the characteristics of fatty acid-mediated ER stress and apoptosis in primary hepatocytes; (2) To determine whether α-linolenic acid, an unsaturated fatty acid, could provide protection against the cell death induced by stearic acid; (3) once the beneficial actions of α-linolenic acid were confirmed, this study inquiryed whether these effects were mediated via modification of the ER stress process with specific attention given to the role of GRP78, GRP94 expression and induction of CHOP.

## 2. Materials and methods

### 2.1 Materials and cells

Rat hepatocytes were isolated from newborn (1-day-old) Sprague-Dawley rats using the modified two-step collagenase perfusion technique as previously described [22]. Freshly prepared hepatocytes were seeded at a density of 2 × 10^5 ^cells/well on 24-well multidishes precoated with rat tail collagen type I solution in Williams Medium E containing 5% of fetal calf serum, 100 nM insulin, 2.5 μg/ml amphotericin B, 0.1 mg/ml gentamicin, 30 nM Na_2_SeO_3_, and 0.1 μM dexamethasone (Sigma-Aldrich, St. Louis, MO). Calf serum and amphotericin B were present for the first 24 h then omitted.

Cell culture materials and routine chemicals were obtained from Sigma (Oakville, ON, Canada) or Fischer Scientific (Nepean, ON, Canada). Primary antibodies were obtained from Stressgen (Victoria, ON, Canada) unless otherwise stated.

### 2.2. Incubation of primary hepatocytes

Cultured hepatocytes at 80-90% confluence, were incubated with stearic acid (250 μmol/l) for up to 16 h. Hepatocytes were also incubated with thapsigargin (5 μmol/l). Further incubations were also performed in which hepatocytes were incubated with stearic acid (250 μmol/l) in the absence or presence of α-linolenic (150 or 250 μmol/l) for up to 16 h.

### 2.3. Measurement of cell viability and death

Cell viability and death were assessed as described previously by measurement of the enzymatic conversion of the yellow tetrazolium salt 3-(4, 5-dimethylthiazol-2-yl)-2, 5-diphenyltetrazolium bromide (MTT) into purple formazan and the release of lactate dehydrogenase (LDH) from lysed cells, respectively [23]. Primary rat hepatocytes were stained with Hoechst 33342-propidium iodide (HPI) to assess cell death by apoptosis and necrosis, respectively [24]. Specifically, apoptotic cells were distinguished as those with characteristic nuclear fragmentation and intense staining of condensed chromatin. Propidium iodide does not enter cells with intact plasma membranes, however, after entering damaged apoptotic or non-apoptotic cells it stains nuclear DNA pink. One thousand, randomly distributed nuclei were counted per sample and were scored as morphologically normal, apoptotic and necrotic using an inverted fluorescence microscope (Axiovert 25, Zeiss) set at excitation and emission wavelengths of 365 and 397 nm, respectively.

### 2.4. Measurement of caspase-3 activation

Caspase-3 activity was evaluated using a DEVD-NucView™ 488 Caspase-3 substrate kit (Biotium Inc., Cambridge, UK). In the presence of active caspase-3 enzyme, the substrate dissociates from its bound fluorogenic DNA-binding dye and the latter binds to DNA and emits fluorescence. Caspase-3 was detected by microscopic examination and also by adapting the kit for microplate fluorescence reading. For this, cells were incubated with 50 μL of 5 μmol/L DEVD-NucView™ 488 Caspase-3 substrate for 30 min. Fluorescence was measured in a microplate reader (Cary Eclipse, Varian Inc.) set at wavelengths of 490 nm excitation and 520 nm emission.

### 2.5. Western blot analysis

Western blot analysis was performed as described in detail previously [25]. Membranes were incubated with antibodies against glucose-regulated protein 78 (GRP78; Stressgen, Victoria BC, Canada), glucose-regulated protein 94 (GRP94; Stressgen, Victoria BC, Canada), CCAAT/enhancer-binding protein homologous protein (CHOP; Santa Cruz Biotechnology, Santa Cruz, CA), and actin (Sigma). Proteins were detected with horseradish peroxidase-conjugated secondary antibodies (Amersham Pharmacia Biotech, Piscataway, NJ) and an enhanced chemiluminescence reagent (Pierce, Rockford, IL). Density was quantified using a UVP Bioimaging system (Upland, CA).

### 2.6. Statistical analysis

Results are expressed as mean ± standard error of the mean (S.E.M.) for n independent observations as indicated. Statistical differences between mean values of groups have been determined using one way analysis of variance (ANOVA) followed by a Dunnett's post-significance test for comparison of multiple means using the SPSS version 11.5. The level of significance was set at P < 0.05.

## 3 Results

### 3.1. Stearic acid causes significant cellular death of hepatocytes - protection by α-linolenic acid

**Figure 1:**
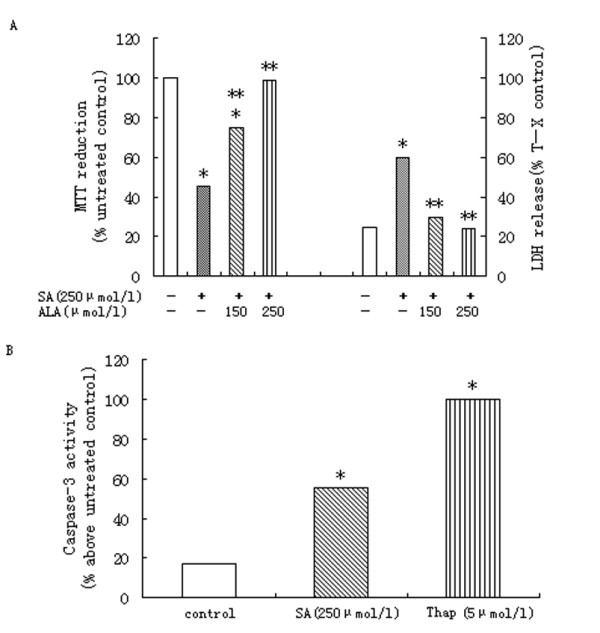
**Effects of SA on primary rat hepatocytes**. (A) Treatment with stearic acid (SA) for 16 h produced significant and concentration-dependent effects on MTT reduction and LDH release. Data represent mean ± S.E.M., n = 5,*P < 0.05 vs. control (0 μmol/l stearic acid), **P < 0.05 vs. stearic acid-only cells. (B) Treatment of primary rat hepatocytes with 250 μmol/L SA produced a significant increase in activity of caspase-3. For comparison, the effects of thapsigargin(Thap; 5 μmol/l)are also shown. Data represent mean ± S.E.M., n = 5,*P < 0.05 vs. control (0 μmol/l stearic acid), **P < 0.05 vs. stearic acid-only cells.

Incubation of subconfluent cultures of the hepatocytes with 250 μmol/l stearic acid for 16 h produced a significant loss of cell viability as demonstrated by decreased reduction of MTT and increased LDH release (Figure 1A). The mode of cell death observed at this concentration of stearic acid was a combination of apoptosis and necrosis as determined using a combination of HPI staining (Figure 1B). However, co-incubation of primary rat hepatocytes with 250 μmol/l stearic acid and 150 or 250 μmol/l α-linolenic acid restored cell viability to levels observed in untreated cells (Figure 1A).

**Figure 2:**
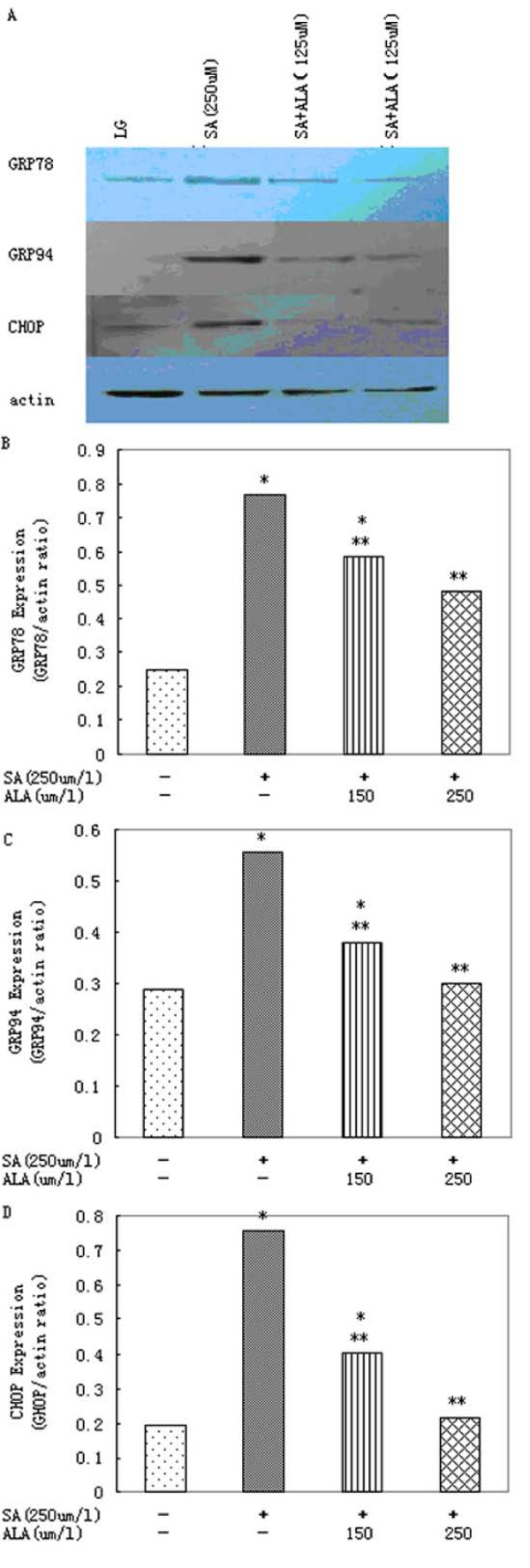
**α-Linolenic acid protects primary rat hepatocytes against ER stress induced by stearic acid**. (A)Western blot and (B) densitometric analysis demonstrating the reduction of stearic acid (SA)-induced GRP78 expression by 150 or 250 μmol/l α-linolenic acid (ALA) after 16 h. (A)Western blot and (C) densitometric analysis of GRP94 expression after 16 h incubation of cells with 250 μmol/l stearic acid(SA) in presence of 150 or 250 μmol/l α-linolenic acid (ALA). (A)Western blot and (D) densitometric analysis demonstrating the reduction of stearic acid (SA)-induced CHOP expression by 150 or 250 μmol/l α-linolenic acid (ALA) after 16 h. Data represent mean ± S.E.M., n = 5, *P < 0.05 vs. LG, low glucose control (0 μmol/l stearic acid), **P < 0.05 vs. stearic acid-only cells.

Stearic-mediated apoptosis of primary rat hepatocytes coincided with a significant increase in caspase-3 activation (Figure 2B). Thapsigargin as well as stearic acid increased fluorescence in the caspase 3 assay confirming activation of apoptosis pathways (Figure 2B).

### 3.2. α-Linolenic acid reduces ER stress mediated by stearic acid

Stearic acid produced a significant increase in the expression of markers of ER stress. After 16 h incubation with stearic acid, increased levels of GRP78, GRP94 and CHOP were also detected (Figure 2).

In the presence of α-linolenic acid, ER stress mediated by stearic acid was significantly reduced. Co-incubation of hepatocytes with 250 μmol/l stearic acid and 150 or 250 μmol/l α-linolenic acid produced a significant reduction in levels of GRP78, GRP94 and CHOP after 16 h (Figure 2).

### 3.3. Effects of α-linolenic acid on primary rat hepatocytes death mediated by thapsigargin

**Figure 3:**
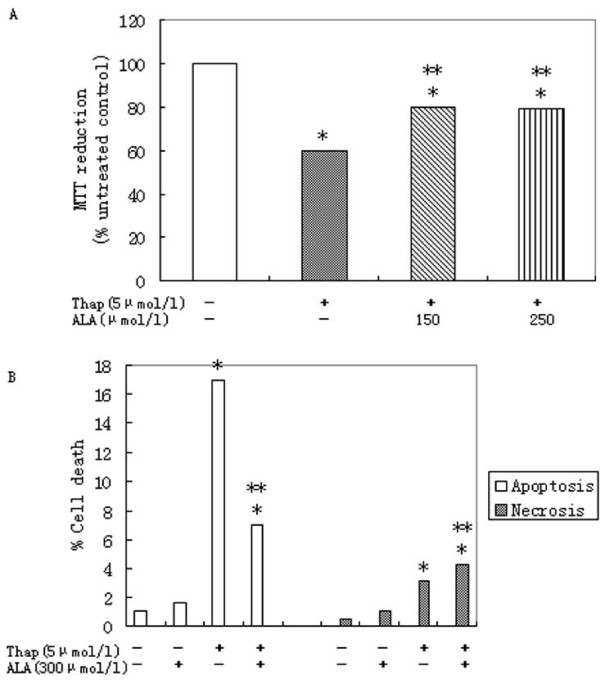
**α-Linolenic acid protects against dysfunction and apoptosis of primary rat hepatocytes induced by thapsigargin**. Relative cell death(A) and relative caspase-3 expression(B) treated with 5 μmol/l thapsigargin (Thap) for 16 h in presence of 150 or 250 μmol/l α-linolenic acid (ALA). Data represent mean ± S.E.M., n = 5, *P < 0.05 vs. LG, low glucose control set to 1 (0 μmol/l stearic acid), **P < 0.05 vs. thapsigargin-only cells.

Incubation of primary rat hepatocytes with 5 μmol/l thapsigargin for 16 h causes significant increases in cell death (Figure 3A). α-Linolenic acid at concentrations of 250 μmol/l, was able to increase cell viability significantly (Figure 3A) -- an effect which was reflected by a significant reduction in the caspase-3 of apoptotic cells (Figure 3B).

### 3.4. Effects of α-linolenic acid on ER stress induced by thapsigargin

**Figure 4:**
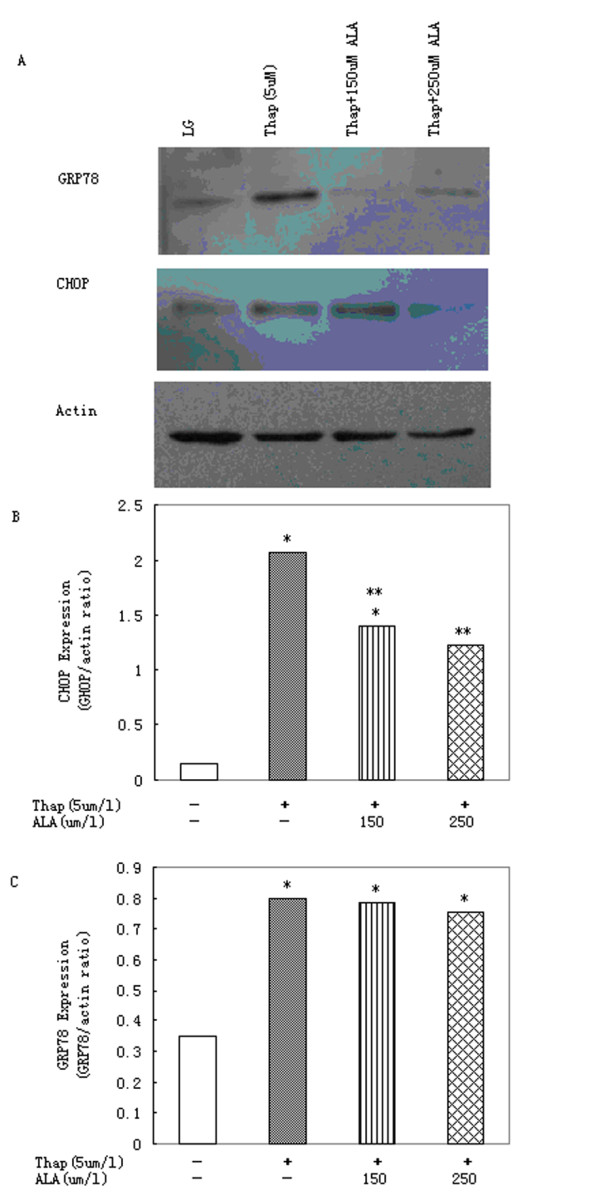
**α-Linolenic acid protects primary rat hepatocytes against ER stress induced by thapsigargin**. (A)Western blot image and densitometric analysis of (B) GRP78 and (C) CHOP expression in cells treated with thapsigargin (Thap; 5 μmol/l) in presence of increasing concentrations of α-linolenic acid (ALA) for 16 h. Data represent mean ± S.E.M., n = 5, *P < 0.05 vs. LG, low glucose control (0 μmol/l stearic acid), **P < 0.05 vs. thapsigargin-only cells.

Incubation of primary rat hepatocytes with 5 μmol/l thapsigargin for 16 h produced a significant increase in levels of CHOP (Figure 4A, C). α-Linolenic acid, at concentrations of 250 μmol/l, was able to reduce the increase in CHOP levels produced by 5 μmol/l thapsigargin (Figure 4A, C). But, neither concentration was able to reduce GRP78 expression mediated by thapsigargin (Figure 4A, B).

## 4. Discussion

The delivery and accumulation of lipids in non-adipose tissues leads to cellular dysfunction and death. This phenomenon, termed lipotoxicity, has been implicated in the pathogenesis of diabetes, cardiac failure and NAFLD [13,26,27]. Disruption of ER homeostasis and activation of the UPR has been observed in murine models of obesity, cardiac dysfunction and NAFLD [13,15,28].

Increased free fatty acids, in particular long chain saturated fatty acids, induce ER stress, activate the UPR and promote cell death in a number of cell types, including hepatocytes [29,26-32]. Thus, impairments in ER function appear to contribute to the pathogenesis of several diseases and to cellular impairments associated with lipotoxicity. The present study was undertaken to begin to examine the mechanisms that link saturated fatty acids to ER stress, In this study, we report that (1) stearic acid causes significant cell death in primary rat hepatocytes. (2) stearic acid causes a significant degree of ER stress in primary rat hepatocytes. (3) α-linolenic acid protects primary rat hepatocytes against stearic acid lipotoxicity by reducing ER stress and apoptosis. The effects of stearic acid were similar to those observed using generated by saturated dietary free fatty acids. α-Linolenic acid was also able to reduce cellular dysfunction and apoptosis caused by thapsigargin -- one recognised inducer of ER stress. Thapsigargin causes apoptosis in many other cells such as neuronal cells, pancreatic beta-cells and cardiomyocytes [33-35]. The mechanism underlying the necrotic cell death produced by thapsigargin is still not clear. It is possible that by protecting cells against thapsigargin-induced apoptosis, α-linolenic acid alleviated primary rat hepatocytes damaged.

Recent evidence has linked saturated fatty acid-induced apoptosis to the activation of JNK, pro-apoptotic Bcl-2 proteins, Bim and Bax, and the mitochondrial apoptotic pathway in liver cells [36]. Long-chain saturated fatty acids also induce the ER-associated pro-apoptotic factor, CHOP, which suggests that disruption of ER homeostasis may also be directly linked to apoptosis. In several cell types, including liver, co-supplementation of oleate and palmitate reduces palmitate-mediated ER stress and apoptosis [30,37,38]. Toward this end, the ability of long chain saturated fatty acids to activate the ER-associated caspase-3 was examined.

In this study, we have demonstrated for the first time that ER stress produced by stearic acid in primary rat hepatocytes can be significantly reduced by α-linolenic acid, an unsaturated fatty acid. We also report that the protection afforded by an unsaturated fatty acid involves a reduction of ER stress. The mechanism involves a reduction in the raised levels of caspase-3 and CHOP associated with stearic acid, however the results of our investigation appears to rule out a protective mechanism mediated by GRP78 as α-linolenic acid did not significantly affect levels of this chaperone molecule whereas levels of CHOP were significantly reduced.

To summarize, the results presented here suggest that unsaturated fatty acids such as α-linolenic acid may be able to offer protection of hepatocytes against the lipotoxicity of saturated fatty acids such as dietary stearic acid and nutrient overload associated with obesity and NAFLD.

## Competing interests

The authors declare that they have no competing interests.

## Authors' contributions

YZ conceived, designed and coordinated the work, as well as prepared the manuscript. LD was involved in the co-design of the work as well as the draft of the manuscript. XY carried out analytical work, HS carried out analytical work and contributed in drafting the manuscript. LZ carried out analytical and statistical analysis. All authors read and approved the final manuscript.
